# Live volumetric (4D) visualization and guidance of *in vivo* human ophthalmic surgery with intraoperative optical coherence tomography

**DOI:** 10.1038/srep31689

**Published:** 2016-08-19

**Authors:** O. M. Carrasco-Zevallos, B. Keller, C. Viehland, L. Shen, G. Waterman, B. Todorich, C. Shieh, P. Hahn, S. Farsiu, A. N. Kuo, C. A. Toth, J. A. Izatt

**Affiliations:** 1 Department of Biomedical Engineering, Duke University, Durham, NC 27708, USA; 2 Department of Ophthalmology, Duke University Medical Center, Durham, NC 27710, USA

## Abstract

Minimally-invasive microsurgery has resulted in improved outcomes for patients. However, operating through a microscope limits depth perception and fixes the visual perspective, which result in a steep learning curve to achieve microsurgical proficiency. We introduce a surgical imaging system employing four-dimensional (live volumetric imaging through time) microscope-integrated optical coherence tomography (4D MIOCT) capable of imaging at up to 10 volumes per second to visualize human microsurgery. A custom stereoscopic heads-up display provides real-time interactive volumetric feedback to the surgeon. We report that 4D MIOCT enhanced suturing accuracy and control of instrument positioning in mock surgical trials involving 17 ophthalmic surgeons. Additionally, 4D MIOCT imaging was performed in 48 human eye surgeries and was demonstrated to successfully visualize the pathology of interest in concordance with preoperative diagnosis in 93% of retinal surgeries and the surgical site of interest in 100% of anterior segment surgeries. *In vivo* 4D MIOCT imaging revealed sub-surface pathologic structures and instrument-induced lesions that were invisible through the operating microscope during standard surgical maneuvers. In select cases, 4D MIOCT guidance was *necessary* to resolve such lesions and prevent post-operative complications. Our novel surgical visualization platform achieves surgeon-interactive 4D visualization of live surgery which could expand the surgeon’s capabilities.

Minimally-invasive microsurgical techniques have revolutionized various surgical disciplines, including ophthalmic, vascular, pediatric, orthopedic, plastic, and neurosurgery^1^. However, microsurgery is more technically demanding than general surgery^2^ and requires a combination of stereoscopic vision through an operating microscope, visio-spatial skills, and precise dexterity that results in a steep learning curve^3^,^4^. In ophthalmic microsurgery—the most frequently performed human surgery in the United States^5^—the surgeon must identify sub-millimeter tissues with subtle contrast and accurately judge micro-architectural alterations. These requirements are crucial to minimize residual tissue damage and maximize efficiency, two prominent factors when assessing microsurgical efficacy^4^. Unfortunately, the basic principles of the operating microscope have not changed since the 1930’s, and its limited depth perception and fixed visual perspective impede important feedback to the surgeon^6^. The introduction of new imaging technologies which are superior or complementary to the conventional operating microscope could improve real-time feedback and dramatically expand surgical capabilities.

Tomographic imaging technologies, such as computed tomography (CT), magnetic resonance imaging (MRI), and ultrasound, allow non-invasive cross-sectional visualization of subsurface human anatomy that is inaccessible with conventional microscopy^7^,^8^,^9^. However, intraoperative applications of CT, MRI, and ultrasound suffer from increased complexity/cost, limited resolution, incompatibility with surgical instruments, and/or the inability to image in real-time^10^,^11^,^12^,^13^. Recent work on near-infrared (NIR) optical imaging demonstrated potential for guiding open-body and laparoscopic surgeries^14^. However, most current NIR intraoperative applications require exogenous contrast agents and are restricted to *en face* two-dimensional (2D) imaging.

Efficient real-time visualization of the large datasets generated with the aforementioned modalities can be also challenging especially in the operating suite. During image-guided general surgery, wall-mounted displays and more recently developed head-mounted displays can relay important feedback to the surgeon^14^,^15^. These techniques however are not generally applicable in microsurgery where the surgeon’s visual field is constrained by the operating microscope oculars. Instead, microscope-integrated heads-up displays (HUD) are necessary to relay data directly into the surgeon’s oculars, but current implementations are limited to monocular projection of 2D images^16^,^17^,^18^. Alternatively, others employed a stereo camera to record and project the view through the operating microscope onto a stereo external monitor^19^. All these previous intraoperative feedback mechanisms however were limited to visualization of 2D images, and more recent techniques for manipulation and visualization of volumetric data^20^ are not currently compatible with live human microsurgery.

Optical coherence tomography (OCT) employs NIR illumination and interferometric detection for volumetric tissue imaging^21^. Because OCT imaging achieves micron-scale resolution and does not require contact with the tissue surface, it has become essential in diagnosing human eye pathology^22^,^23^ and is quickly gaining prominence in vascular^24^,^25^,^26^, pediatric^27^, and cancer^28^ applications. High-resolution non-invasive tissue evaluation with OCT was also adopted for various intrasurgical applications^29^, including eye^16^,^30^,^31^,^32^,^33^,^34^ and breast^35^ human surgery. However, all of these previous intraoperative applications were limited to 2D planar imaging (B-scans) of live surgery, which resulted in difficulties and delays associated with identifying a single 2D plane of interest within a 3D surgical field and interpreting it in its correct spatial context. For example, previously reported systems could not image continuous instrument motion since only a portion of the instrument was visualized within the B-scan plane and instrument motion was not constrained within that plane. This limitation rendered guidance of surgical maneuvers with these systems unfeasible. Another study demonstrated a custom Spectral Domain intraoperative OCT system with graphics processing unit (GPU) compatible software for real-time volumetric imaging and rendering, but this system was limited to *ex vivo* surgical phantoms due to its sub-optimal sensitivity and image quality^36^. Additionally, many of the previous systems did not provide instantaneous OCT feedback to the surgeon during live surgery, which is also a pre-requisite for image-guided surgery.

In this work, we report on the translation of a microscope-integrated OCT (MIOCT) system that achieves, to our knowledge, the first four-dimensional (4D: volumetric imaging through time) real-time presentation of live human microsurgery to the surgeon. Our 4D MIOCT system is able to image, record, and render the 3D ophthalmic surgical field at up to 10 volumes per second. In comparison to previous intraoperative imaging modalities, 4D MIOCT achieves more comprehensive live surgical visualization (live 3D imaging versus live 2D imaging using the operating microscope and previous intraoperative OCT applications) with micron-scale spatial resolution in all three-dimensions. The 4D data is rendered in real-time using custom GPU based software and relayed to the microscope oculars using a novel dual-channel stereoscopic HUD to provide immediate feedback to the surgeon. We report that 4D MIOCT improved (with statistical significance) the performance of select depth-based maneuvers in mock surgical trials compared to maneuver guidance with the operating microscope alone. During imaging of 48 human eye surgeries, we demonstrates 4D MIOCT identification of previously unrecognized tissue micro-architectural alterations during common surgical maneuvers, and 4D MIOCT-guided resolution of lesions that were invisible under conventional surgical illumination.

## Results

### 4D MIOCT system

**Figure 1:**
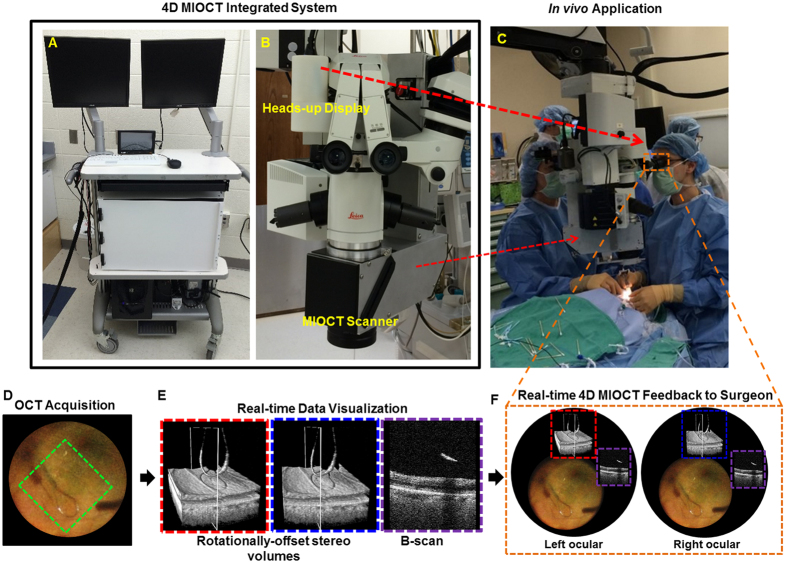
Integrated 4D MIOCT system and real-time feedback to the surgeon. (**A**) Photograph of the 4D MIOCT system portable cart that housed the OCT laser, interferometer, electronics, and processing computer. (**B**) Photograph of MIOCT scanner, operating microscope, and heads-up display (HUD). The HUD and MIOCT scanner integrated seamlessly into microscope. (**C**) Photograph of system in use during human surgery. The microscope-integrated design enabled OCT data acquisition during *live* surgery. (**D**) 4D MIOCT acquisition with the scan (green box) oriented to the axis of the surgical instrument. The MIOCT scan axis could be arbitrarily rotated and shifted laterally to scan the surgical site of interest. (**E**) Rotationally-offset stereo MIOCT volumes and B-scans rendered in real time. (**F**) Projection of the 4D MIOCT data and other supporting surgical data into the oculars for real-time OCT feedback to the surgeon.

The integrated 4D MIOCT microsurgical imaging system is shown in Fig. 1. A portable cart housed the custom OCT hardware, computer, and software-user interface (Fig. 1A). The OCT sample arm design employed a customized mechanical enclosure for seamless integration into a Leica M844 ophthalmic surgical microscope (Fig. 1B)^31^ to enable OCT data acquisition during *live* surgery without disturbing surgical visualization through the operating microscope. To achieve fast volumetric OCT imaging, an ultra-high speed swept-frequency source centered at 1040 nm (Axsun Technologies; Billerca, MA) was used, and the interferograms were digitized at 800 MS/s and processed with custom software on a graphics processing unit (GPU). The volumetric data was rendered to a two dimensional image using optimized ray casting, edge enhancement, and depth-based shading algorithms^37^. The system was capable of acquiring and processing OCT volumes at up to 10 volumes per second, although in human surgical trials volume rates of 3.33 Hz and 2 Hz with higher sampling density were selected for retinal and anterior eye surgery, respectively. A detailed description of the optical design and software is provided in the Methods section.

### 4D MIOCT data acquisition and visualization

Surgery-tailored MIOCT scan control methods and visualization techniques were developed to facilitate image acquisition and feedback (Fig. 1D–F). During surgery, the operator could rotate the MIOCT scan axis to align the B-scan direction to a particular maneuver, tool, or region of interest, and used a manual tracking module (Supplementary Fig. S1) to relocate the OCT scanning site within the surgical field in real-time. The volumetric rendering perspective was also adjustable by the operator in real-time. Mixed mode volumes, in which only the B-scan of interest was densely sampled and averaged while the rest of the data was sparsely sampled, were used to preserve a fast volumetric rate and high-quality B-scans. The acquired volumetric data was displayed in four different formats: rendered volumes, B-scans (cross-sections), maximum intensity projection (MIP) *en face* views, and two rotationally-offset volume renders for stereoscopic visualization. The surgeon visualized the stereo volumes and B-scans during surgery (detailed below) while the volumes, MIPs, and B-scans were projected on a wall-mounted display in the operating suite and a computer monitor for feedback to the surgical team.

To provide immediate stereo 4D MIOCT visualization to the surgeon with surgeon control of the volumetric rendering perspective, we utilized a novel heads-up display (HUD) integrated into the operating microscope (Fig. 1B)^38^. The dual-channel stereoscopic HUD allowed simultaneous projection of the rotationally-offset MIOCT volumes into the surgical oculars using a single micro-display (Fig. 1F) (optical design detailed in the Methods section). The location of data projected within the oculars using the HUD was controlled by the operator to ensure that the surgical field was not obstructed. A foot-operated joystick allowed the surgeon to change the rendering orientation of the MIOCT volumes projected with the HUD. This feature enabled the surgeon to inspect the surgical field with an adjustable volumetric visual perspective. A B-scan was also projected into the surgical oculars for real-time visualization by the surgeon, and the location of the displayed B-scan within the volume was denoted by a white rectangle overlaid on the volume render (Fig. 1E). The OCT operator controlled the location of the B-scan with guidance from the surgeon or surgical assistant and could turn on/off the B-scan and volume view or add the MIP if desired by the surgeon. Alternatively, the surgeon could control the location of the entire volume and displayed B-scan by rotating the patient’s eye or repositioning the microscope laterally.

### Preliminary 4D MIOCT *ex vivo* studies

**Figure 2:**
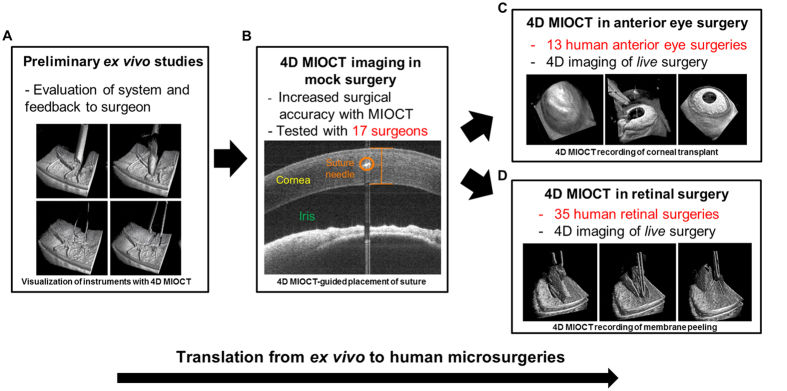
Translation of prototype 4D MIOCT system from *ex vivo* studies to human microsurgeries. (**A)** Preliminary studies were performed to evaluate 4D MIOCT performance, visualization of microsurgical instruments, and feedback to the surgeon using the HUD. (**B**) 4D MIOCT-guided surgical maneuvers were quantitatively evaluated in mock surgical trials involving 17 surgeons and surgeons-in-training. (**C**,**D**) The utility of *in vivo* 4D MIOCT imaging was evaluated in 13 human anterior eye surgeries (**C**) and 35 human retinal surgeries (**D**).

The integrated 4D MIOCT system was tested with a replica of the operating microscope in a wet laboratory setting before translation into the human operating suite (Fig. 2A). MIOCT visibility of commonly used ophthalmic surgical instruments (including different instrument gauges) was characterized during retinal surgery in *ex-vivo* porcine whole eyes (Supplementary Table S1). Simulated surgical maneuvers were also performed in cadaveric porcine eyes and human donor corneas to test MIOCT volumetric visualization of anterior segment and retinal surgery at different volume rates and sampling densities. Using a 5 × 5 mm and 10 × 10 mm field of view for retinal and anterior eye surgery, respectively, the volumetric frame rate was varied between 1–10 volumes per second by varying the number of A-lines per volume to determine the fastest volume rate that would still allow sufficient lateral resolution for human surgical visualization. The optimal scan parameters selected were 300 A-lines/B-scan and 100 B-scans for retinal surgery, and 500 A-scans/B-scan and 100 B-scan for anterior eye surgery, which resulted in 3.33 Hz and 2 Hz volume rates, respectively. However, if desired for particular applications in future studies, higher volume rates with similar sampling density can be achieved over smaller fields of view.

### 4D MIOCT quantitatively enhances performance of depth-based microsurgical skills in mock surgical trials

Because 4D MIOCT offers improved depth perception compared to the operating microscope, we hypothesized that our system could enhance the performance of depth-based microsurgical maneuvers. We tested this hypothesis for two conventional microsurgical skills: suture placement at target depths within tissue and control of instrument positioning relative to tissue (Fig. 2B).

**Figure 3:**
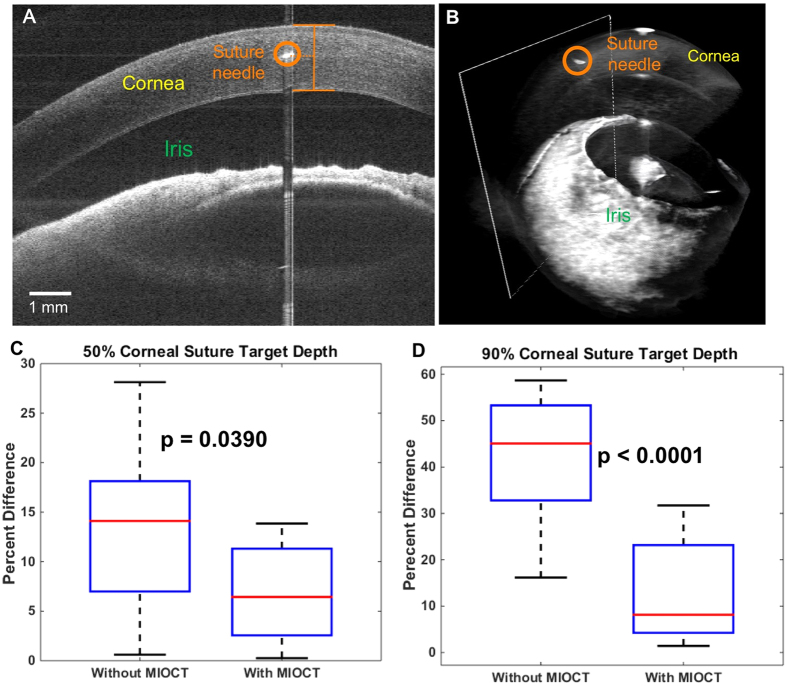
4D MIOCT increases suture placement accuracy in mock surgical trials. A group of 14 surgeons-in-training were asked to perform corneal suture passes at two target depths with and without 4D MIOCT guidance during simulated surgery in cadaveric porcine eyes. (**A**) Representative B-scan located at the point of maximal needle depth. The needle, cornea, and iris are labeled in orange, yellow, and green, respectively. (**B**) Corresponding volumetric image. The B-scan (**A**) location is denoted by white rectangle. In this trial, the surgeon inserted the suture needle at a 44% corneal depth, 6% away from the 50% target depth. The cross-sectional view allowed accurate grading of the suture needle placement within corneal tissue. (**C**,**D**) Box plots summarizing suture placement with and without MIOCT guidance for 50% (**C**) and 90% (**D**) target depths, respectively. The data is plotted in percent difference from the target depth; closer to 0% difference is more accurate. At both target depths, the surgeons achieved increased suture placement accuracy with MIOCT.

To test suture placement with 4D MIOCT guidance, a group of 14 ophthalmic surgeons-in-training were recruited from Duke University ophthalmology residents to perform suture passes at target depths within *ex vivo* porcine corneas^39^. The subjects were randomized into two groups: one that used MIOCT for guidance, and one without MIOCT (N = 7). Each subject was asked to perform a corneal suture pass at both 50% and 90% depth and was allowed up to three trials before performing two gradable attempts for each depth. During the trials, only the group with MIOCT guidance was allowed direct depth visualization of the suture needle within corneal tissue using volumes and B-scans after passing the suture (Fig. 3A,B) (see Methods section for more details).

MIOCT guidance improved suture placement accuracy at both 50% and 90% target depths (Fig. 3C,D). The average difference from the target depth with MIOCT and without MIOCT was 7.07% (SD = 4.99%) and 12.69% (SD = 8.31%), respectively, for the 50% target depth. For 90% target depth the average difference with MIOCT and without MIOCT was 12.31% (SD = 10.03%) and 43.63% (SD = 12.83%), respectively. The performance improvement with MIOCT guidance for both 50% and 90% suture placement target depths was statistically significant with P = 0.0390 and P < 0.0001, respectively.

**Figure 4:**
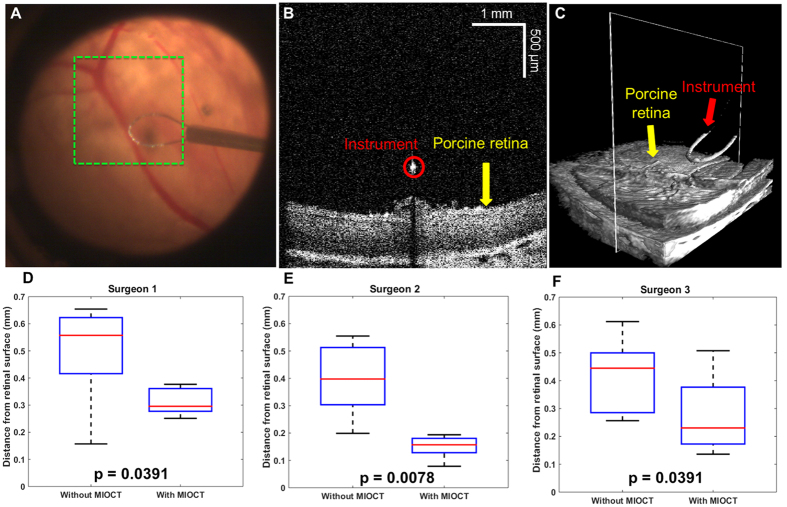
4D MIOCT improves the control of instrument placement relative to tissue in mock surgical trials. Three ophthalmic surgeons were asked to place their instrument as close to retina as possible without contacting tissue during simulated porcine eye retinal surgery. Each surgeon performed the maneuver 8 times with and without 4D MIOCT guidance. (**A**) Porcine retina as viewed through the operating microscope. The green dashed box denotes the 5 × 5 mm MIOCT lateral field of view. (**B**) B-scan located at the point of the instrument’s closest proximity to the retinal surface. The instrument and retina are labeled in red and yellow, respectively. The cross-sectional view allowed accurate grading of the instrument/retina distance. (**C**) Corresponding volumetric image with the B-scans (**B**) location denoted by the white rectangle. In this trial, the surgeon placed the instrument 214 μm above the retinal surface. (**D**–**F**) Box plots summarizing instrument placement relative to the retinal surface with and without MIOCT guidance for all 3 ophthalmic surgeon participating in the study. Improved visual feedback with MIOCT guidance allowed each surgeon to place their instrument closer to the retinal surface (closer to zero without contacting) than without MIOCT guidance.

To test control of instrument placement with MIOCT guidance, 3 ophthalmic surgeons were recruited from Duke University ophthalmology retinal surgery fellows. The surgeons were asked to place a surgical instrument (nitinol surgical flex loop (Alcon; Ft. Worth, TX)) as close to the retinal surface as possible without contacting tissue during simulated retinal surgery in cadaveric porcine eyes. Each surgeon performed the maneuver 8 times without MIOCT guidance and 8 times with MIOCT guidance (N = 8) after 3 practice trials. When performing the maneuvers under MIOCT guidance, the surgeon was given the option of visualizing the MIOCT data in real-time either through the microscope oculars (using the HUD) or on a monitor adjacent to the microscope (Fig. 4A–C), both with surgeon control of the volume perspective (see Methods section for more details).

MIOCT guidance improved the control of instrument placement relative to tissue of all 3 surgeons in the study (Fig. 4D–F). The average instrument to retina distance without MIOCT and with MIOCT was 500 μm (SD = 173 μm) versus 312 μm (SD = 48 μm) for surgeon 1, 398 μm (SD = 128 μm) versus 150 μm (SD = 39 μm) for surgeon 2, and 416 μm (SD = 130 μm) versus 275 μm (SD = 133 μm) for surgeon 3, respectively. The performance improvements for surgeons 1, 2, and 3 were statistically significant with P = 0.0391, P = 0.0078, and P = 0.0391, respectively.

### 4D MIOCT translation into the human operating suite

To determine the utility of 4D MIOCT *in vivo*, the system was tested in 48 human microsurgeries, including 35 retinal and 13 anterior eye surgeries (Fig. 2C,D). All human participant studies were performed after obtaining informed consent under protocols approved by the Duke University Health System Institutional Review Board. All *in vivo* volumes and B-scans were processed in real-time and did not require additional post-processing for presentation. Additional details on maximum permissible light exposure to the patient and imaging protocols are provided in the Methods section.

### 4D MIOCT imaging of human retinal surgery

Retinal microsurgery involves restoration of macro and micro-architectural retinal alterations that arise from pathologic conditions. In one such condition, a proliferation of abnormal tissue membrane on the surface of the retina (epiretinal membrane) contracts and causes visual distortion and loss of central vision. Macular holes (full thickness retinal breaks) and lamellar holes (partial thickness retinal breaks) can also result from a combination of traction from collagen fibers in the vitreous gel, contraction of surface membranes and of the inner retinal surface basal lamina (internal limiting membrane). Microsurgical forceps and/or scrapers are used to peel these pathologic and/or native membranes to relieve the underlying retinal contraction and close the retinal defect.

MIOCT imaging was performed on patients undergoing standard 3-port vitrectomy with three surgeons at a single site. Inclusion criteria for imaging included macular surgery for membrane removal, macular hole or lamellar hole closure, and/or retinal detachment repair. MIOCT imaging was attempted in a total of 35 retinal surgeries. To evaluate success of MIOCT imaging, a trained technician graded the acquired data based on the identification of pathology of surgical interest in MIOCT B-scans or volumes in concordance with preoperative diagnosis. A summary of results organized by pathology is provided in Table 1. Note that multiple pathologies could be present in one surgical case. The pathology of interest was successfully visualized with MIOCT in 93% of attempted cases. MIOCT imaging was unsuccessful in 3 retinal detachment cases because of insufficient field of view (1 case) and inability to focus the OCT beam on retina due to the severity of the detachment (2 cases).

**Figure 5:**
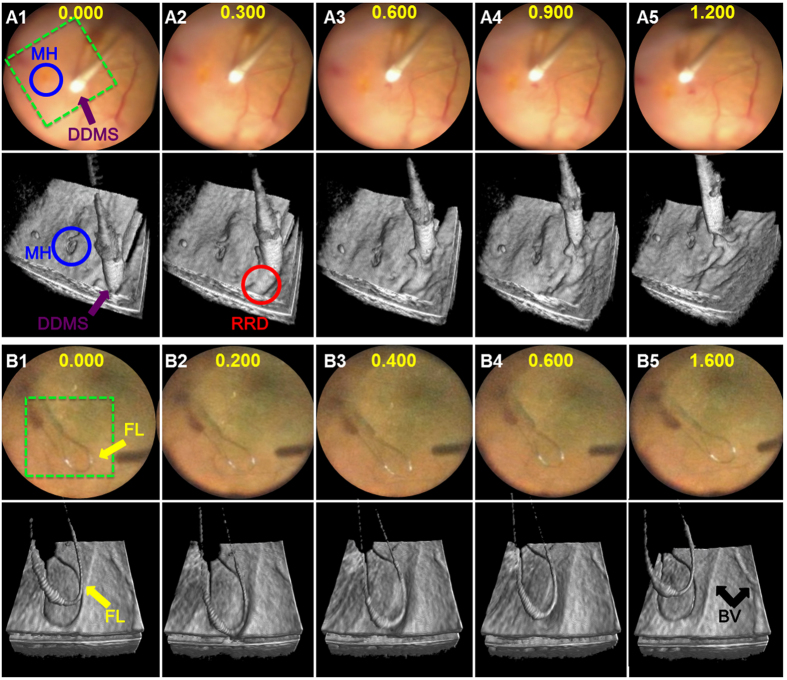
4D MIOCT reveals previously unidentified volumetric tissue deformation during human retinal brushing. 4D MIOCT was performed during retinal brushing to initiate peeling of a pathologic membrane in human retinal surgery. (**A**) Excerpts from a 4D MIOCT recording showing retinal brushing with a diamond dusted membrane scraper (DDMS) (purple) around a macular hole (MH) (blue) (Movie S1). The corresponding frames from the surgical camera used to record the view through the operating microscope are shown above the MIOCT data. The macular hole is more readily identifiable in the MIOCT volumes. In addition, volumetric retinal deformation during the brushing maneuver and residual retinal deformation (RRD) (red) after the maneuver was only visible in the MIOCT data. (**B**) Excerpts from a 4D MIOCT illustrating retinal brushing with a microsurgical flex loop (FL) (yellow) and elevated blood vessels (BV) (black) (Movie S2). Compared to (**A**), the flex loop is less abrasive and results in less overall retinal deformation and no residual deformation. The volumetric frames rates for (**A,B**) were 3.33 and 5.0 volumes/second, respectively. Time stamps are in seconds (yellow numbers). The green dashed box denotes the lateral MIOCT field of view. The volumetric MIOCT field of view was 3 × 5 × 5 mm.

4D MIOCT revealed previously unrecognized micro-architectural alterations and instrument-tissue interactions that occurred during conventional surgical maneuvers. Excerpts from a 4D MIOCT recording (Fig. 5A and Supplementary Movie S1) show visualization of a diamond dust-coated membrane scraper brushing the retina around a full-thickness macular hole and producing a transient indentation of the inner retinal contour adjacent to the hole. Corresponding frames from a surgical camera that records the surgeon’s view through the operating microscope are shown above each MIOCT volume. The depth of the macular hole (partial versus full-thickness) and tissue deformation by the surgical instrument could not be identified using the conventional view through the operating microscope alone. After brushing was completed, residual retinal deformation was noted but was not as prominent compared to during instrument contact, which emphasized the need for real-time imaging during live maneuvers. 4D MIOCT imaging was also performed during retinal brushing with the microsurgical nitinol flexible loop (yellow arrow) (Fig. 5B and Supplementary Movie S2). The MIOCT volumes illustrate the instrument-tissue interaction, including deformation of the retinal surface across the area of instrument contact. Both this interaction and elevation of retinal blood vessels above the retinal surface (black arrows) were not visible via conventional microscope viewing. The proximity of the instrument to retina could also be determined more precisely in 4D MIOCT volumes compared to the surgical microscope view. In contrast to the diamond-dusted scraper, the loop appeared to be less abrasive and resulted in transient focal retinal depression during time of contact but not afterward.

**Figure 6:**
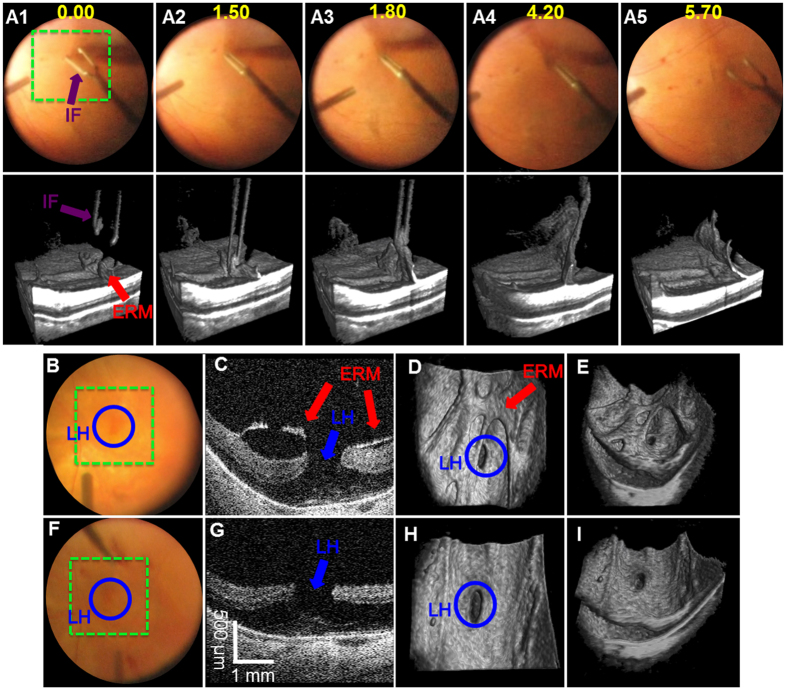
4D MIOCT enhanced visualization during removal of human pathologic translucent membranes. 4D MIOCT was performed during peeling of epiretinal membranes (ERM) in human retinal surgery. (**A**) Excerpts of a 4D MIOCT recording illustrating grasping and peeling of an ERM (red) with surgical intraocular forceps (IF) (purple) (Movie S3). The maneuver is visualized in the surgical camera frames (top row) and volumes (bottom row). The ERM flap used to initiate the peeling is more readily identifiable in the MIOCT data. Additionally, the angle and membrane tension during the ERM peel are more appreciable in the MIOCT images. (**B**–**E**) show the camera frame (**B**), B-scan (**C**), and volumetric renderings (**D,E**) of ERM (red) around a macular hole (MH) (blue) before peeling in a separate surgery. The exact axial proximity of the ERM to underlying healthy retina is only directly visible in the B-scan (**C**) and volumes (**D,E**). The complex 3D ERM microarchitecture could be inspected in volumetric MIOCT data from different perspectives (**D,E**). (**F**–**I**) shows similar views of the macula after ERM peeling. The volumetric frame rate was 3.33 volumes/second. Time stamps are in seconds (yellow). The green dashed box denotes the lateral MIOCT field of view. The volumetric MIOCT field of view was 3 × 5 × 5 mm.

4D MIOCT also improved real-time visualization during the pathologic tissue removal from the retinal surface. Removal of these membranes and underlying basal lamina (internal limiting membrane) is challenging through the operative microscope because they are translucent, hundreds-of-micrometers thick, and often require staining^40^,^41^ to facilitate their identification. 4D MIOCT enabled clear visualization of the elevated portion of the membrane (red arrows) that was grasped with surgical forceps (purple arrows) to initiate peeling (Fig. 6A, and Supplementary Movie S3) without tissue staining. Important parameters that the surgeon must consider to minimize retinal damage during peeling—including membrane tension, associated deformation of the retinal surface, and angle of the membrane relative to retina—were also better appreciated with 4D MIOCT than through the conventional microscope view. Moreover, the membrane architecture and its proximity to underlying retina was consistently visible with 4D MIOCT irrespective of visible light endoscopic illumination angle and position, which was required only for conventional microscope viewing.

In addition to live volumetric visualization, the surgeon’s ability to view 4D MIOCT B-scans and static volumes at various rendering perspectives via the HUD provided important feedback. In a separate membrane peeling case, 4D MIOCT images (Fig. 6C–E) allowed enhanced visualization of partially separated surface membrane tissue (red arrows)—which was attached at the margin of a partial thickness macular hole (blue arrow)—compared to the limited conventional *en face* view (Fig. 6B). The different perspectives of the 3D micro-architecture of the membrane and retina were controlled by the surgeon in real-time with a foot-operated joystick. The altered viewpoint in Fig. 6E improved visualization of the membrane attachment at the margin of the hole in contrast to the viewpoint in Fig. 6D. After tissue removal, 4D MIOCT volumetric images and B-scans showed that the membrane was successfully removed and that retinal tissue (photoreceptors) was present at the base of the hole (below blue arrow), verifying that the hole had remained partial thickness and did not progress to full thickness during surgery (Fig. 6G,H). The surgeon-controlled viewpoint also augmented the view of retinal vessels elevated across the inner surface and the residual hole (Fig. 6H,I).

### 4D MIOCT imaging of human anterior eye surgery

Anterior eye surgeries, including cataract surgery and corneal transplants, are among the most commonly performed surgeries in the United States. Cataracts are caused by protein build up in the native ocular lens that leads to reduced visual acuity. In cataract surgery, the native lens is fragmented using an ultrasonic probe or femtosecond laser and replaced with an artificial intraocular lens. Corneal transplantation may be required to treat a number of conditions, including injury from trauma, corneal edema, or tissue scarring. During corneal transplantation, at least a portion of the patient’s diseased cornea is replaced with a donor graft. Proper graft placement reduces the risk of rejection and results in improved visual acuity for the patient. However, the graft/native cornea interface is not readily visible in conventional surgery using the operating microscope due to its limited depth visualization. Additionally, corneal edema can limit the surgeon’s view of the graft under standard operating microscope illumination^42^.

4D MIOCT imaging was performed on patients undergoing anterior eye surgery with two surgeons at a single site. Inclusion criteria included full corneal transplantation (penetrating keratoplasty), partial thickness corneal transplantation (Descement’s stripping automated endothelial keratoplasty (DSAEK)), and cataract surgery. MIOCT imaging was attempted in 13 anterior segment surgeries. Successful MIOCT imaging was defined as clear visualization of all the surgical sites of interest in B-scans or volumes, and grading was performed by a trained technician. A summary of results organized by surgical procedure is provided in Table 2. Note that multiple procedures could be performed during one surgery. Successful MIOCT imaging was achieved in 100% of the attempted cases.

**Figure 7:**
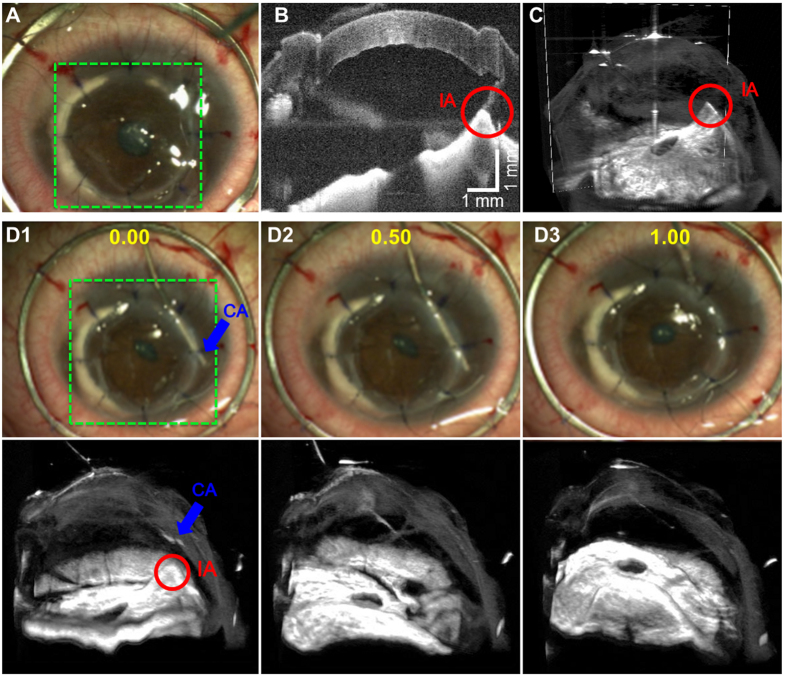
4D MIOCT-guided identification and resolution of abnormal adhesion of iris to the graft/cornea interface during a human corneal transplantation. 4D MIOCT guidance was necessary to treat the lesion the lesion since it could not be visualized top-down through the operating microscope. (**A–C**) show the microscope view (**A**), B-scan (**B**), and volume (**C**) at the time during which the surgeon identified the abnormal iris adhesion (IA) to cornea/graft interface (red) in the MIOCT data. The white rectangle in the volume denotes the location of the B-scan. (**D**) Excerpts of 4D MIOCT recording during treatment of the lesion (Movie S4). Using 4D MIOCT for localization guidance, the surgeon was able to direct a cannula (CA) (blue) and inject viscoelastic between the iris and corneal graft to release the adhesion. Further evaluation using MIOCT revealed resolution of the adhered iris with clear intervening space between iris and cornea. The volumetric rate for (**D**) was 0.5 volumes/second. Time stamps (yellow) are in seconds. The green dashed box denotes the lateral MIOCT field of view. The volumetric MIOCT field of view was 6 × 10 × 10 mm.

4D MIOCT imaging was *necessary* to reveal and guide the treatment of select lesions during a full corneal transplantation. During the procedure, 4D MIOCT revealed an abnormal adhesion (red circle) of the iris to the graft/cornea interface after securing the graft (Fig. 7B,C). This adhesion was not visible with the operating microscope (Fig. 7A) and could have led to post-operative complications—such as wound leakage, local corneal endothelial loss, increased inflammation, and glaucoma—if not resolved. Using 4D MIOCT for localization and guidance, the surgeon directed a cannula (blue arrows) to inject viscoelastic and release the iris (Fig. 7D and Supplementary Movie S4). The surgeon then verified the resolution of the abnormal adhesion by identifying clear intervening space between the iris and cornea on the 4D MIOCT data (Fig. 7D time: 1.00 s).

**Figure 8:**
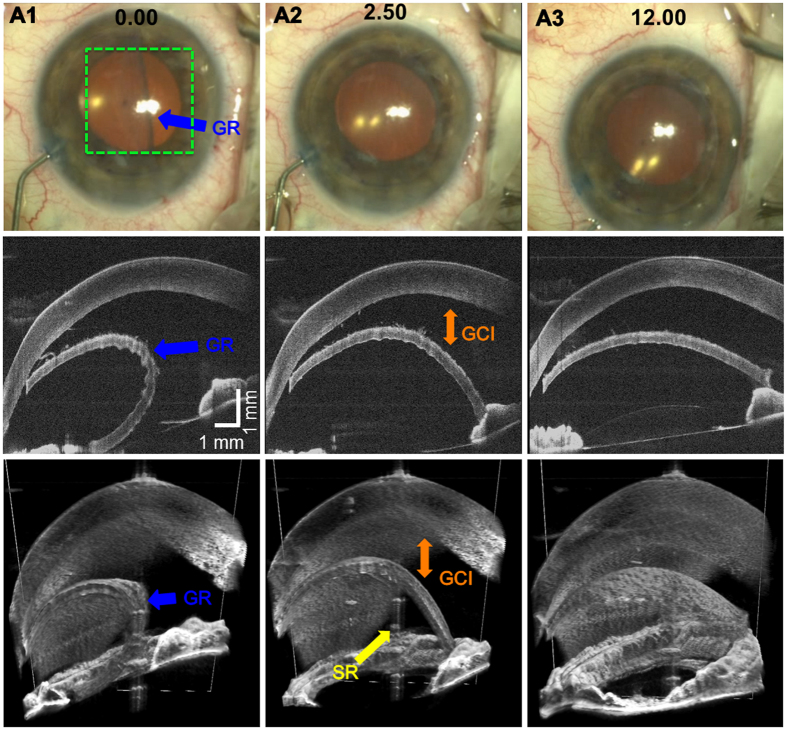
4D MIOCT-guided unfolding of graft below native cornea during human partial thickness corneal transplantation. 4D MIOCT imaging was necessary to determine the axial distance between the graft and native cornea, which was invisible through the operating microscope. (**A**) Frames from the surgical camera (top) and excerpts from a 4D MIOCT recording shown in B-scans (middle) and volumes (bottom) during graft insertion and unfolding (Movie S5). As the graft (GR) (blue) unfolded, the surgeon used 4D MIOCT data to monitor the graft/cornea interface (GCI) (orange) and to ensure graft/cornea apposition. The volumetric rate for was 0.5 volumes/second. An image artifact due to specular reflection (SR) (yellow) from the corneal apex is present in the middle of the volumes. Time stamps (black) are in seconds. The white rectangle in the volumes denotes the location of the B-scan. The green dashed box denotes the lateral MIOCT field of view. The volumetric MIOCT field of view was 6 × 10 × 10 mm.

4D MIOCT imaging also helped guide the placement of the corneal graft in a partial thickness corneal transplantation. During the DSAEK procedure, only the posterior 10% cornea is replaced with a donor graft; therefore, the surgeon must insert, unfold, and manipulate the graft beneath the remaining native cornea. Figure 8 and Supplementary Movie S5 show a 4D MIOCT recording of the graft unfolding after insertion with the corresponding surgical camera frames. While the folded graft (blue arrows) could be identified in the operating microscope, the axial proximity of the graft to native cornea was only visible with MIOCT. The surgeon then injected air to unfold the graft and compress the intervening space between the graft and cornea. MIOCT was used during the maneuver to monitor graft placement and confirm graft/host apposition. Failure to eliminate graft/host interface fluid would result in post-operative graft detachment and need for additional surgery to reattach the graft.

## Discussion

While tomographic modalities can image subsurface anatomy, most intraoperative implementations suffer from low resolution, instrument incompatibility, and/or the inability to image in real-time^7^,^8^,^9^,^10^,^11^,^12^,^13^. OCT is a real-time volumetric modality that uses interferometric detection to achieve micron-scale, structural imaging of tissue. Since its inception in 1991^21^, OCT has become the gold standard diagnostic modality for preoperative evaluation and postoperative assessment of surgical endpoints in most retinal and many corneal procedures. The development of handheld OCT (HHOCT) also allowed imaging of supine, anesthetized patients and the first OCT imaging at predefined time points of ophthalmic surgeries; however, these probes required gross displacement of the microscope away from the surgical field and could not be used during live surgery^43^,^44^,^45^. Our previous MIOCT work laid the foundation for real-time tomographic feedback to the surgeon and achieved B-scan imaging of live surgery for the first time^46^,^47^. Since, similar research-grade and commercial systems has been developed, but all are limited to B-scans for real-time imaging and therefore cannot comprehensively image the dynamic 3D surgical field^16^,^48^,^49^.

We described a 4D MIOCT system that, together with a custom HUD and surgeon control of visualization, offers a novel integrated 4D imaging platform that enhances the feedback available to the surgeon. For example, surgeons currently rely on indirect visual cues such as shadows or instrument deflection to estimate the pressure exerted on tissue with their instruments. Our 4D MIOCT system allows surgeons to precisely determine the instrument-tissue distance and volumetric tissue deformation during instrument contact (Fig. 5). Such new information may become critical for evaluating the surgeon’s instrument control and residual tissue damage caused by instrument contact. Additionally, dyes such as indocyanine green^40^ are often used to facilitate identification and dissection of translucent pathologic membranes through the operating microscope, but a recent study correlated their use with decreased visual acuity for patients in the first postoperative year^41^. We demonstrated that 4D MIOCT could help identify and guide the dissection of such membrane without the need for dyes (Fig. 6). Additionally, 4D MIOCT was necessary to identify and guide the treatment of select lesions, and to monitor the volumetric localization of grafts relative to native corneal tissue in select anterior eye surgeries (Figs 7 and 8). This improved surgical feedback afforded by our system may facilitate development of future ocular surgical therapies based on volumetric, micron-scale visualization of surgery, including precise removal or movement of layers of retinal or corneal tissue and the placement of biomedical devices^50^ (such as retinal prostheses) in the proper location relative to native tissues.

Compared to high-resolution MIOCT B-scan imaging alone^49^,^18^,^17^,^51^,^52^, fast volumetric imaging may improve OCT visualization of complete surgical maneuvers and 3D alterations of tissue anatomy. B-scan imaging of complete surgical maneuvers requires the surgeon to restrict his/her motion to the B-scan axis—which is unfeasible in an *in vivo* setting—or automated instrument tracking to continuously reposition the B-scan axis during the maneuver. The latter proposition however would add cost and complexity to the system, and recently demonstrated techniques have been restricted to anterior segment mock surgery^53^,^54^. In our study, fast volumetric OCT imaging relaxed these constraints and facilitated imaging of transient tissue deformation (Figs 5 and 7) and surgical maneuvers (Figs 6 and 8). Moreover, volumetric imaging may provide to the surgeon lateral context that more closely resembles the view through the operating microscope compared to B-scans alone, which could also facilitate real-time interpretation of the OCT data.

Preoperative and postoperative OCT assessment of the microsurgical field is crucial to analyze the progression of pathologies and evaluate surgical success. Our 4D MIOCT system successfully imaged either the pathology or surgical site of interest in 95% of human surgeries (Tables 1 and 2) and provided comparable diagnostic information *during* live surgery. This intraoperative feedback enables surgeons to continuously monitor regions of interest throughout the surgical procedural progression and can potentially alter the surgeon’s decision-making to optimize outcomes. We expect this benefit to be immediately impactful in the ophthalmic surgical community and are currently conducting in-depth studies examining the utility of 4D MIOCT to monitor microsurgeries beyond those described here.

Our mock surgery results make a compelling case that 4D MIOCT guidance can improve the accuracy of important microsurgical skills such as suture placement at target depths within tissue and control of instrument positioning relative to sensitive tissue (Figs 3 and 4). In both cases, surgeons exploited the augmented depth perception provided by MIOCT to improve their surgical accuracy. Although cross-institutional large-scale studies will be required to evaluate the long-term impact of MIOCT on surgical training^39^ and performance, the results presented here demonstrate that for maneuvers requiring precise dexterity in the depth dimension MIOCT can improve the performance of surgeons with varying levels of experience.

While our initial clinical target for 4D MIOCT guided surgery was ophthalmic microsurgery due to the expertise of our team, the underlying intrasurgical imaging technology has much broader potential application to many surgical specialties. Live 3D OCT is equally applicable to imaging neural tissue in the retina or at a craniotomy site, whether in the hands of an ophthalmic surgeon, an otolaryngologist, or a neurosurgeon. Intraoperative 3D surgical visualization with direct surgeon control of the viewpoint of the tissue volume revolutionizes the surgeon’s ability to act on real-time changes in the surgical field regardless of the tissue or specialty. Additionally, quantitative metrics derived from OCT volumes—including attenuation coefficients of tissue and thickness measurements—have been demonstrated to differentiate normal tissue from tumor or high-grade malignancy in the kidney, prostate, vulva, breast and brain in animal models or after excision during surgical pauses^28^,^55^,^56^,^57^,^58^. An expected future application of 4D MIOCT could be the implementation of such quantitative measurements to differentiate normal and malignant tissue at a microsurgical margin and guide human tumor resection in real-time rather than evaluating the excised tissue during pauses in surgery.

Although 4D MIOCT presents substantial advantages over current microsurgical visualization techniques, there are important limitations that must be addressed. Namely, the maximum volumetric frame rate (~10 volumes per second) in the presented prototype system was inherently limited by the sweep rate of the laser. Fast volumetric frame rates are important to mitigate motion artifacts resulting from rotation/translation of the patient’s eye and to capture fast surgical maneuvers. Research-grade swept-source OCT systems achieving volumetric rates of >20 volumes/second have been demonstrated, but these require significant development before use in large-scale human subject studies and cannot be integrated into operating microscopes^59^,^60^,^61^,^62^. Nonetheless, rapid commercial development of swept-source lasers is ongoing, and near-future increases in the line-scan and volumetric imaging rates may be anticipated using lasers with faster sweep rates and laser sweep buffering schemes^63^. Additionally, the optical properties of current ophthalmic surgical instruments (e.g. forceps, picks, surgical blades) are not optimized for OCT imaging (Table S1). Many instruments are made from metallic alloys that provide stability and precision required to perform microsurgery but cast shadows on underlying tissues^64^. Moreover, highly reflective instruments may result in saturation when the instrument is oriented orthogonally to the OCT optical axis. Recent work demonstrated OCT-optimized surgical tools that are compatible with our MIOCT system, which achieve sufficient OCT visibility while minimizing shadowing and saturation artifacts^16^.

In summary, we have reported on the first micrometer-scale 4D visualization of human microsurgery. The 4D MIOCT prototype improved the accuracy of widely-applicable surgical maneuvers during mock surgery. I*n vivo* 4D MIOCT results from 48 human surgeries enhanced the visual feedback available to the surgeon. Comprehensive 4D visualization of microsurgery with our system can improve current ophthalmic surgery and may catalyze the development of novel surgical techniques in the future.

## Methods

### Design of 4D MIOCT scanner

The MIOCT scanner optics comprised a collimating lens followed by an afocal magnifying telescope (Thorlabs, Inc.; New Jersey, USA) to match effective numerical aperture (NA) of the OCT system to the NA of the surgical microscope^31^ (Supplementary Fig. S2). A turning mirror on a kinematic mount redirected and folded the optical path and was used to ensure that the OCT scan location was centered within the surgical field. A dichroic mirror with a reflectivity cutoff of 990 nm coupled the OCT and microscope optical paths. The OCT beam was then focused by the operating microscope objective. To adjust the OCT focus plane relative to the microscope, the OCT fiber terminator before the collimator was placed on a micrometer-based linear translator (Thorlabs, Inc.; Newton, New Jersey). Either the OCT operator or surgical assistant actuated the micrometer to change the beam vergence prior to the objective and refocus the OCT. For retinal imaging, a surgical contact lens (Alcon; Ft. Worth, TX) was placed on the patient’s cornea to relay the OCT beam to the retinal plane. For anterior segment imaging, the OCT beam was directly focused on the subject’s cornea. The axial and lateral resolutions of the MIOCT system were 7.8 μm and 14 μm, respectively. The lateral imaging range was limited to about 5 × 5 mm by the patient’s ocular pupil in retinal surgery and 10 × 10 mm by the optical design in anterior segment surgery. The system achieved imaging at a depth-scan (A-line) rate of 100 kHz with high sensitivity (102 dB peak signal to noise standard deviation ratio), up to 7.4 mm depth imaging range, and 4.9 mm −6 dB signal falloff. The anti-reflection (AR) coating of the commercial microscope objective was not optimized for near IR, which resulted in sub-optimal (65%) transmission of the OCT light and an associated reduction in imaging sensitivity compared with using lenses with an appropriate anti-reflective coating.

### GPU-accelerated volumetric rendering with lighting, edge, and depth-enhanced ray casting

Custom software enabled real time acquisition, processing, and rendering of volumetric data sets acquired at 100 kHz line rates (Supplementary Fig. S3)^37^. The software was written in C/C++ and was comprised of three concurrent threads: a data collection thread, a data processing and rendering thread, and a display thread. The data collection thread communicated with the digitizer and collected 4000 spectral samples of data for each A-scan. Blocks of 16 B-scans were processed at a time through the use of custom GPU code written in CUDA and executed on a GTX Titan (NVIDIA; Santa Clara, CA). The 4D MIOCT control software provided real time 3-view rendering including full ray-cast volumetric visualization at up to 10 volumes per second. The volumetric view was created using an enhanced, real time, integrated volumetric rendering engine for OCT data that mimics the quality of those produced in commercial post-processing software. The rendering pipeline incorporated high performance volumetric median (3 × 3 × 3 pixels) and 2D Gaussian filtering (5 × 5 pixels), boundary and feature enhancement, depth encoding, and lighting into a ray casting volume rendering model. A CUDA implementation of this enhanced ray casting model generated real time dynamic renders that dramatically improved depth perception and visualization of microsurgical volumetric features. A customizable histogram transfer function also allowed the operator to assign opacity and color based on the voxel intensities to the final volume render. The NVIDIA profiler timing output for a typical high-density scan setting of 2752 (axial) x 300 (lateral) pixels per B-scan is illustrated in Supplementary Fig. S3.

The maximum system latency, defined as the maximum delay between acquisition and display of data from any particular sample location, was governed by the time required to acquire, process, and render that data. As described in ref. 37, our approach to minimize latency was to process data in blocks of 16 B-scans while the next block was being acquired, thus limiting latency to 116.5 ms for our longest B-scans comprising 512 A-scans (86 ms acquisition +30 ms processing/rendering). For B-scans containing less than 512 A-scans, the computational time scaled down linearly with the number of A-scans per B-scan^37^. For repetitive volumetric imaging, the maximum time between updates of data from any particular sample location was given by the total volume acquisition time, which in our system was 0.3 seconds for retinal imaging and 0.5 seconds for anterior segment imaging.

### Design of stereoscopic HUD

The stereoscopic HUD was designed to be mechanically and optically integrated into the surgical microscope (Supplementary Fig. S4)^38^. An organic LED microdisplay (Olightek Co., Ltd.; Kirkland, WA) with high contrast ratio (10,000:1) was relayed to an intermediate imaging plane with a 50 mm focal length imaging lens (Edmund Optics, Inc., Barrington, NJ) and a ~190 mm focal length lens located in the operating microscope binocular head. The intermediate image plane was then relayed to the surgeon’s retina with a 10x microscope eyepiece (Leica Microsystems; Wetzlar, Germany). The optical path of the HUD was coupled into the path of both surgical oculars using two beam splitters in the microscope infinity space. The beam splitters were tilted by 1.5 degrees relative to the HUD optical axis so that only the left half of the microdisplay was projected to the right ocular, while the right half of the microdisplay was projected to the left ocular. Therefore, separate images could be displayed into the two oculars using only one microdisplay. To project stereoscopic OCT data, two rotationally-offset volumetric renders were projected into the left and right ocular. A foot-operated joystick allowed the surgeon to control the rendering perspective of the volumes to facilitate visualization of the surgical field.

### 4D MIOCT mock surgery set up

For anterior segment and retinal porcine eye surgery, fresh enucleated porcine eyes were obtained from a local slaughterhouse, stored at 4 °C, and used within 12–18 hours to minimize corneal edema. For retinal surgery, standard 3-port vitrectomy was utilized: one port was used to insert the infusion line to regulate intraocular pressure, the second port was used to insert a fiber-optic endoilluminator, and the third port was used to insert the surgical instruments. Alternatively, fresh research grade donor corneas were obtained from a local eye bank (Miracles in Sight; Winston-Salem, NC).

### Quantitative studies during mock surgery

For the suture depth study, each group (MIOCT vs. no MIOCT) consisted of 3 first-year, 2 second-year, and 2 third-year residents (N = 7) to remove potential bias from level of training and surgical experience. Both groups performed the gradable attempts without MIOCT feedback. The maximal depth of the needle and suture were determined after acquiring volumetric MIOCT scans encompassing a 7.4 × 10 × 10 mm field of view centered on the pass. To grade the suture placement, B-scans at the point of maximal depth of the suture needles were marked by a masked trained grader.

For the instrument positioning study, the group consisted of 1 first-year and 2 second-year surgical fellows to remove potential bias from level of training. All maneuvers were recorded with MIOCT volumetric scans encompassing a 3 × 5 × 5 mm field of view centered on the surgical instrument. To grade the distance of the instrument to retinal surface, B-scans showing the instrument cross section closest to the retinal surface were marked by a masked grader.

### Grading and statistical analysis

Custom MATLAB software (Mathworks; Natick, MA) was created to grade the suture needle placement. On averaged B-scans at the point of maximal suture needle depth, a masked grader marked the surface of the corneal endothelium, epithelium, and suture needle after refraction correction to compensate for image distortion^65^. The corneal thickness was then identified as the shortest path between the endothelial and epithelial layers that included the marked suture needle position. The percent suture depth was then calculated as the ratio of the depth of the needle of suture (distance from the epithelium to the suture or needle) over the corneal thickness (distance from epithelium to endothelium). The difference between the percent suture depth and target suture depth was then calculated. Commercial software (Meazure, C Thing Software) was used to grade the distance from the instrument to retinal surface in the instrument positioning study. The distance in number of pixels was measured by a masked grader on B-scans at the point of closest instrument proximity to retinal surface and converted to millimeters using a calibration factor.

Statistical comparisons between the groups performing suture placement with and without MIOCT guidance were analyzed with the two-tailed Wilcoxon Rank Sum test, with the null hypothesis that the different samples are from distributions with equal medians. The samples were considered independent since suture placements with and without MIOCT guidance were performed on different cadaveric porcine eyes. P < 0.05 was classified as statistically significant. Statistical comparison between instrument placement trials in porcine retinal surgery performed with and with 4D MIOCT guidance were analyzed with the two-tailed Wilcoxon Sign Rank test, with the null hypothesis that the differences between paired samples come from a distribution with zero median. P < 0.05 was classified as statistically significant.

### 4D MIOCT translation to human subjects

All human participant studies (ClinicalTrials.gov Clinical Trial Identifier: NCT01588041) were performed after obtaining informed consent and in accordance with protocols approved by the Duke University Health System Institutional Review Board. Prior to imaging, informed consent was received from all subjects after explanation of the possible consequences and nature of the study. All methodology and portions of this study followed the tenets of the Declaration of Helsinki and were conducted in compliance with the Health Insurance Portability and Accountability Act. During imaging, MIOCT optical power on the eye was below 1.7 mW and the surgical visible illumination was reduced by 20% to maintain the total irradiance below the maximum permissible exposure for ocular illumination^66^. Unless otherwise specified, retinal MIOCT scans were 3 mm axially and were composed of 300 A-lines/B-scan and 100 B-scans per volume, resulting in a volumetric rate of 3.33 Hz. All anterior segment MIOCT scans were 6–7.4 mm axially and were composed of 500 A-lines/B-scan and 100 B-scan per volume, resulting in a volumetric rate of 2 Hz.

While the functionality of the operating microscope was unchanged after integrating the MIOCT scanner and HUD, the distance between the surgical oculars and microscope objective was increased and resulted in an increase in the height of the surgical oculars relative to the patient. To mitigate potentially negative ergonomic effects due to the height increase, one of the two sets of oculars for surgical assistants was removed before surgery to decrease the height of the main oculars for the surgeon, since only one surgical assistant was required in all cases of interest.

## Additional Information

**How to cite this article**: Carrasco-Zevallos, O. M. *et al*. Live volumetric (4D) visualization and guidance of *in vivo* human ophthalmic surgery with intraoperative optical coherence tomography. *Sci. Rep.* **6**, 31689; doi: 10.1038/srep31689 (2016).

## Supplementary Material

Supplementary Information

Supplementary Movie S1

Supplementary Movie S2

Supplementary Movie S3

Supplementary Movie S4

Supplementary Movie S5

## Figures and Tables

**Table 1:** Identification of pathology with 4D MIOCT in 35 human retinal surgeries.

Preoperative Diagnosis	Number of MIOCT cases	Successful MIOCT visualization of pathology (%)
Lamellar hole	4	4 (100)
Macular hole	12	12 (100)
Epiretinal membrane	17	17 (100)
Retinal detachment	10	7 (70)
Total	43	40 (93)

Successful MIOCT visualization of pathology in 35 retinal surgeries was determined by a trained grader. The tabulated results are organized by pathology; multiple pathologies could be present in a single surgery.

**Table 2:** Identification of surgical site of interest with 4D MIOCT in 13 human anterior eye surgeries.

Procedure	Number of MIOCT Cases	Surgical site of interest	Successful MIOCT visualization of surgical site of interest (%)
Cataract/IOL placement	6	Corneal incision integrity, lens fragmentation, and lens capsule	6 (100)
DSAEK	6	Corneal graft in anterior chamber and graft-host interface	6 (100)
Penetrating keratoplasty	3	Graft-host interface	3 (100)
Total	15	N/A	15 (100)

Successful MIOCT visualization the surgical site of interest in 13 anterior eye surgeries was determined by a trained grader. The tabulated results are organized by procedure; multiple procedures could be performed in a single surgery.
