# Supplementary material for: Live volumetric (4D) visualization and guidance of in vivo human ophthalmic surgery with intraoperative optical coherence tomography
Source: Sci Rep. 2016 Aug 19;6:31689. doi: 10.1038/srep31689 (PMC4990849; doi:10.1038/srep31689)
Supplement: Supplementary Information [file srep31689-s1.pdf]

Title: Live volumetric (4D) visualization and guidance of *in vivo* human ophthalmic surgery with intraoperative optical coherence tomography

Authors: O. M. Carrasco-Zevallos[1], B. Keller[1], C. Viehland[1], L. Shen[1], G. Waterman[1], B. Todorich[2], C. Shieh[2], P. Hahn[2], S. Farsiu[1,2], A.N. Kuo[2], C. A. Toth[2,1], J. A. Izatt[1,2]

Affiliations:

[1] Department of Biomedical Engineering, Duke University; Durham, NC 27708

[2] Department of Ophthalmology, Duke University Medical Center, Durham, NC 27710

Corresponding Author: Joseph A. Izatt; joseph.izatt@duke.edu

Keywords: image-guided surgery, optical coherence tomography, ophthalmic surgery

SUPPLEMENTARY INFORMATION

Supplementary Methods
Supplementary Fig. S1. Manual tracking module characterization.
Supplementary Fig. S2. 4D MIOCT optical design, imaging techniques, and performance.
Supplementary Fig. S3. GPU-accelerated enhanced volumetric rendering flowchart and performance.
Supplementary Fig. S4. Optical design the microscope-integrated stereoscopic HUD.
Supplementary Table. S1. Characterization of MIOCT visibility of ophthalmic surgical instruments.
Supplementary Movie S1. 4D MIOCT of human retinal brushing with a membrane scraper.
Supplementary Movie S2. 4D MIOCT of human retinal brushing with a microsurgical flex loop.
Supplementary Movie S3. 4D MIOCT of enhanced visualization during removal of human pathologic translucent membranes.
Supplementary Movie S4. 4D MIOCT-guided identification and resolution of abnormal adhesion of iris to the corneal/graft interface.
Supplementary Movie S5. 4D MIOCT-guided unfolding of graft below native human cornea.

Supplementary Methods:

Manual tracking module

To compensate for patient motion, a manual tracking module was implemented that allowed the MIOCT operator to relocate the scan location in real-time. The feed from the surgical camera that was coupled to the optical path of the microscope was captured by the MIOCT computer using a frame grabber (Nanjing Magewell Electronics Co., Ltd.; Najing, China). The camera feed was displayed with an overlayed rectangle denoting the OCT lateral scan location within the surgical field. The pixels in the displayed camera images were then assigned a voltage value used to offset the OCT scanning position using an analog summing amplifier. The tracking system was calibrated prior to each imaging session to compensate for lateral and rotational offsets between the camera and MIOCT imaging planes. In real-time operation, the MIOCT operator could relocate the lateral OCT scan location simply by clicking and dragging the rectangle denoting the OCT field of view

to the desired location within the displayed camera images. This feature was useful to realign the scan to a region of interest outside of the surgeon's central view through the operating microscope, or to compensate for misalignment of the OCT when the surgeon repositioned the microscope laterally relative to the patient eye. Additionally, patients were not fully sedated and were capable of small amounts of head and eye motion which could also be compensated for with manual tracking. Although automatic tracking would be ideal, manual tracking was sufficient for the sporadic realignment required to compensate for the infrequent, slow, and relatively predictable motion described above.

The latency of the manual tracking module was characterized in the following manner. The mouse cursor on the tracking module was programmed (LabVIEW, National Instruments; Austin, Tx) to offset OCT scan automatically, eliminating bias introduced by the human operator. The cursor was moved in a sinusoidal manner with varying amplitude using a function generator and the motion of the cursor generated a "tracking" voltage waveform used to reposition the scanning mirror. The input sinusoidal voltage waveform and the output tracking sinusoidal voltage waveform were recorded with a digital oscilloscope and the cross-correlation of these two waveforms yielded the latency of the tracking module (Supplementary Fig. S1A).

Tracking accuracy of the system was measured by mounting a surgical instrument (diamond dusted membrane scraper) on a motorized linear translation stage (Velmex, Inc). An 8x5 mm volumetric scan, composed of 512 A-scans and 128 B-scans, was then acquired centered on the instrument tip and the summed volume projection (SVP) of the volume was calculated and designated as the control image. Next, the translation stage was used to displace the instrument laterally from -5 to 5 mm, in 1 mm increments. At each position, the operators re-centered the OCT field of view on the instrument and the corresponding SVP was calculated, and designated as the "tracked" SVP. The normalized 2D cross-correlations of the tracked and control SVPs were calculated to determine tracking accuracy at varying instrument displacement. The RMS tracking error was calculated and plotted in Supplementary Fig. S1B.

MIOCT characterization of ophthalmic surgical instruments
Surgical instruments evaluated for MIOCT visibility included a 23 gauge diamond dusted membrane scraper (Synergetics, Inc; O'Fallon, MO), 23 and 25 gauge Finesse flex loops (Alcon; Fort Worth, TX), 23, 25, and 27 gauge intraocular forceps (Synergetics, Inc; O'Fallon, MO), a 25 gauge silicone soft tip (Synergetics, Inc; O'Fallon, MO), and a 41gauge subretinal injection needle (Synergetics, Inc; O'Fallon, MO). Each instrument was also imaged with the camera that records the view through the operating microscope and MIOCT volumes composed of 500 A-lines/B-scan and 300 B-scans/volume during simulated porcine eye retinal surgery. The results of the experiments are tabulated below (Supplementary Table S1).

Supplementary figures and tables:

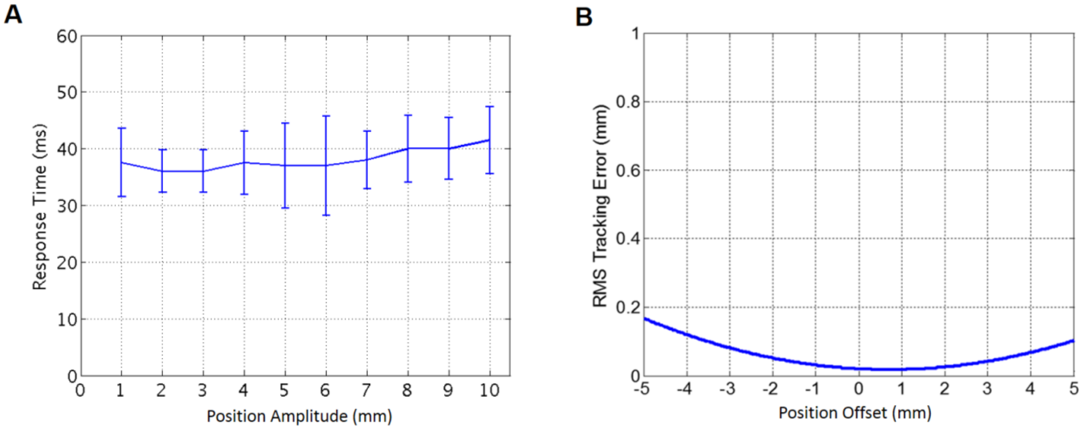

Supplementary Fig. S1. **Latency and tracking accuracy analysis of MIOCT manual tracking module.** The system latency was measured 10 times for different amplitudes of motion, ranging from 1 mm to 10 mm. (**A**) The mean and standard deviation of the latencies for different motion amplitudes. The response time of the system was between 36 ms to 42 ms for amplitudes ranging from .5 mm to 10 mm. (**B**) Tracking error of the manual tracking module. The RMS tracking error was less than .185 mm for a +/- 5 mm displacement range.

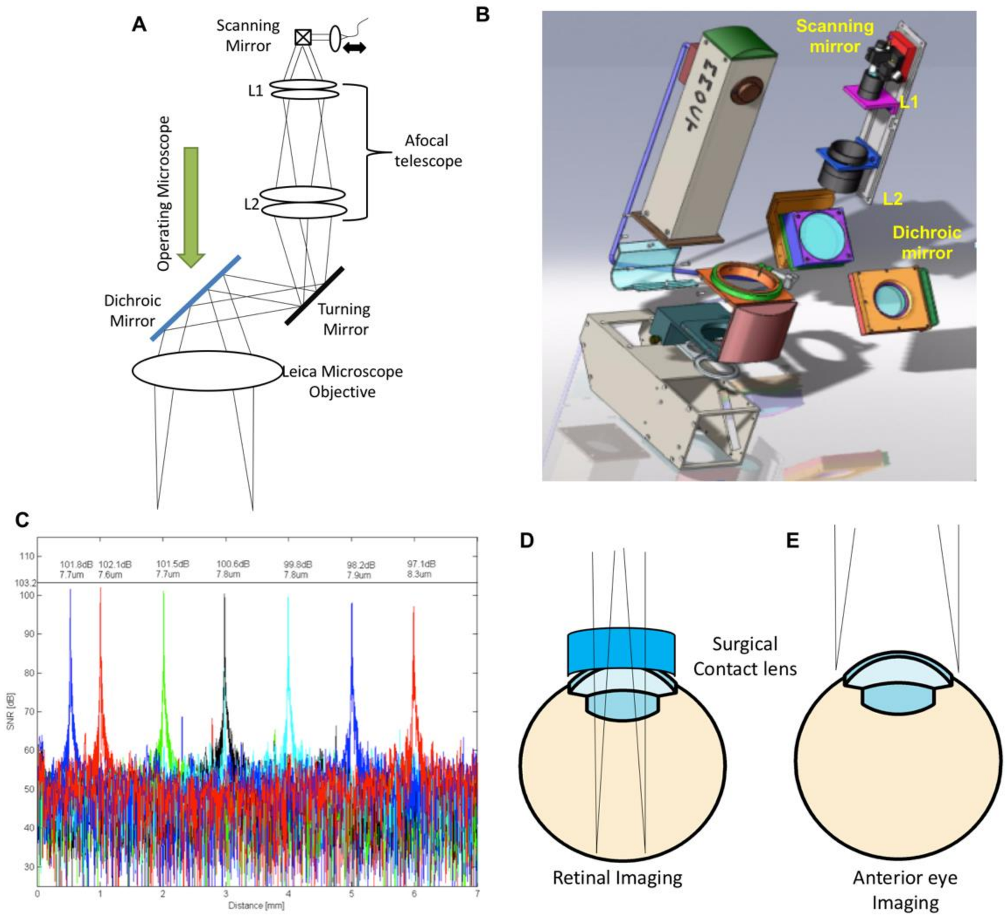

Supplementary Fig. S2. **The optical design and performance of the MIOCT scanner, and description of retinal and corneal imaging configurations.** (**A**) Optical design of the MIOCT scanner that is coupled into the operating microscope using a dichroic mirror. The only lens that is shared by the MIOCT and microscope is the microscope objective. (**B**) Mechanical design of the MIOCT scanner. (**C**) OCT signal-to-noise (SNR) and fall-off performance. The maximum SNR was 102 dB, while the fall off was 4.9 mm. (**D**) Imaging configuration used for retinal imaging. A surgical contact lens was placed on the cornea to visualize retinal structures. (**E**) Anterior segment imaging configuration, in which the MIOCT light was directly focused onto the cornea.

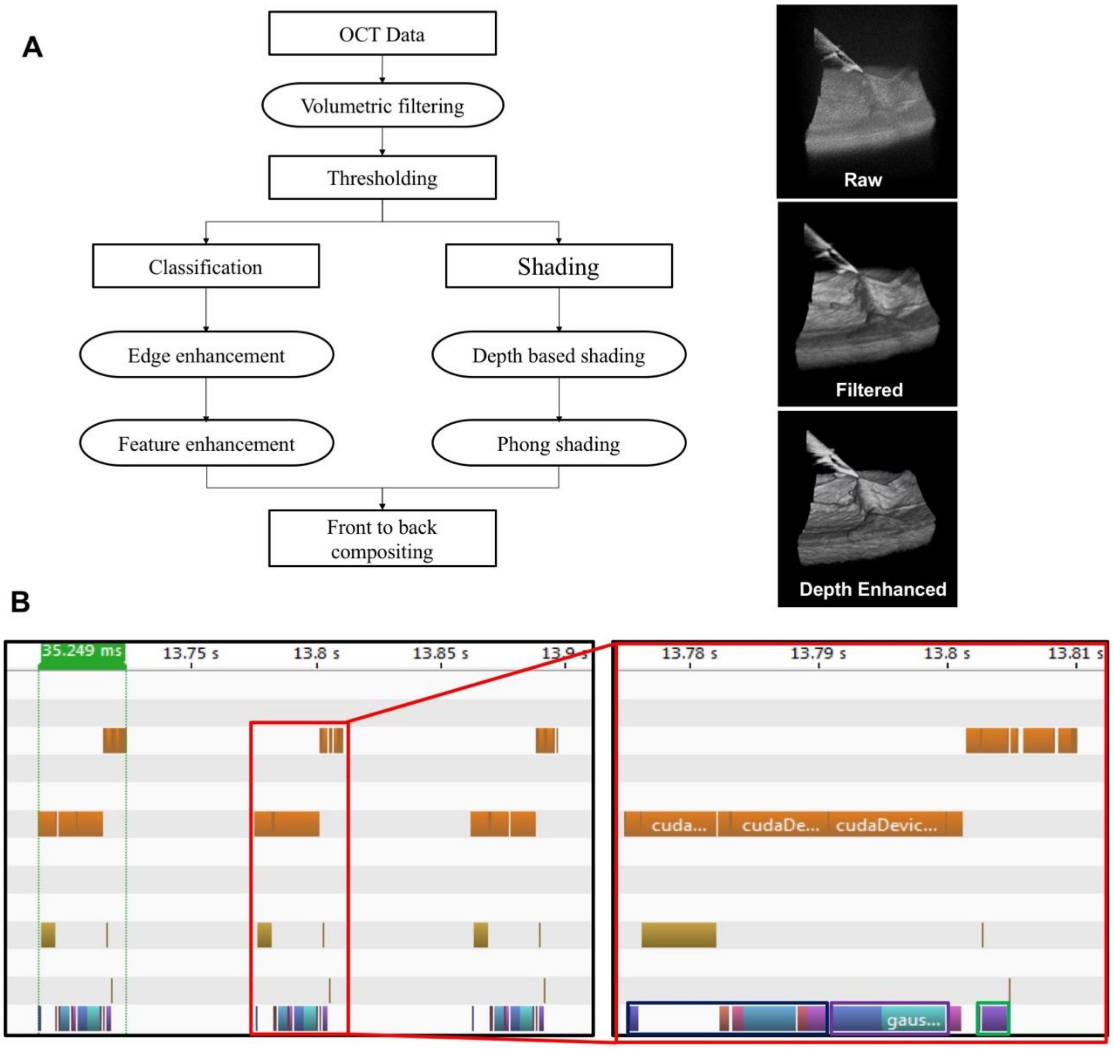

Supplementary Fig. S3. Description of enhanced OCT volumetric rendering steps and timing performance. (**A**) Flow chart for the enhanced ray casting pipeline. Steps in the conventional ray casting are shown in squares while additions are shown in ovals. For each pixel in the output image plane, a ray was projected from the viewpoint, through the image plane, and into the volume. At discrete points in the ray, the voxel values were interpolated and thresholded. Piece-wise shading and classification assigned color and opacity to each voxel. (**B**) Timing diagram showing the acquisition and rendering pipeline for multiple groups of 16 B-scans, with each B-scans composed

of 300 A-scans. The zoomed in view displays the timing characteristics of a single acquisition. Kernels in the acquisition, filtering, and rendering pipelines are highlighted with blue, purple, and green respectively

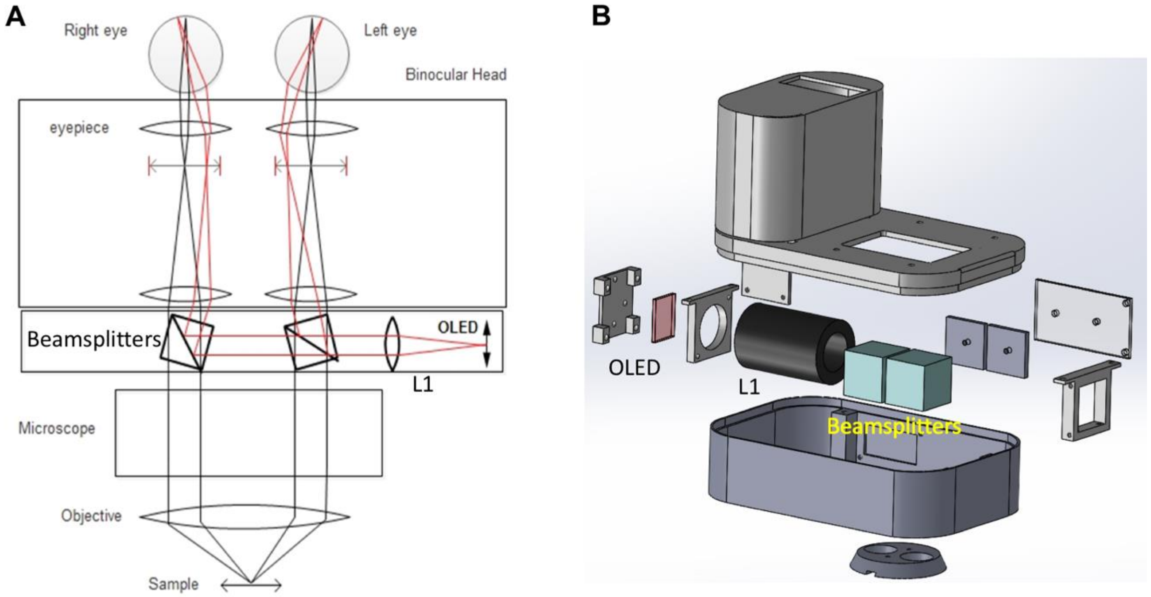

Supplementary Fig. S4. **Optical design of the stereoscopic head-up display (HUD) used to project MIOCT images into the surgical oculars for real-time surgical feedback**. (**A**) An organic LED projector was coupled onto the path of the surgical oculars using beam splitters. These beam splitters were rotated such that the left and right half of the displays were relayed to different oculars. This spatial multiplexing enabled independent projection of different images into the two oculars using one display to reduce the footprint of the module. (**B**) Mechanical design of the HUD.

Supplementary Table. S1. Characterization of the OCT visibility of various ophthalmic surgical instruments. The metallic instruments exhibited excellent OCT visualization but shadowed underlying tissue significantly. However, shadowing appeared to decrease with larger gauge instruments. The nitinol loop instruments exhibited the best compromise between visibility and shadowing. Both anterior and posterior facets of the instrument were clearly visible while underlying tissue shadowing was minimal. The silicone cannula was well visualized with OCT and exhibited minimal shadowing under the tip. Additionally, the tip of diamond duster silicone scraper was highly visible in OCT while shaft was not and the instrument shadowed tissue significantly. Lastly, the 41 gauge subretinal needle was poorly visualized with OCT and only the tip was identifiable in the volume.

Instrument	23g Forceps	25g Forceps	27g Forceps	23g Loop	25g Loop	25g Soft Tip Cannula	25g Scraper	41g Needle
Operating Microscope	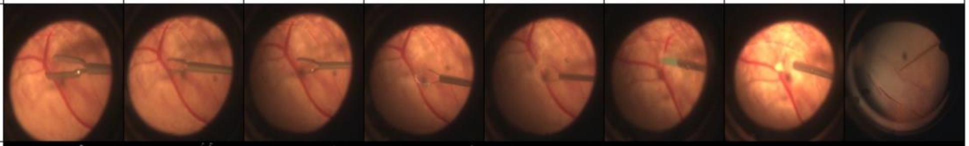							
MIOCT Volume	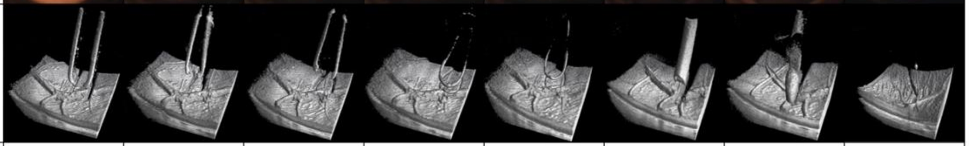							
Company	Synergetics	Synergetics	Synergetics	Alcon	Alcon	Synergetics	Synergetics	Synergetics
Material	Stainless steel	Stainless steel	Stainless steel	Nitinol	Nitinol	Silicone	Diamond dusted silicone	Polymide
Utility	Membrane peeling	Membrane peeling	Membrane peeling	Peel initiation	Peel initiation	Injection and aspiration	Peel initiation	Subretinal injection
OCT Visibility	Excellent	Excellent	Excellent	Sufficient, entire loop is not visible	Sufficient, entire loop is not visible	Excellent	Excellent tip visibility, minimal shaft visibility	Minimal visibility, only needle tip is visible
Shadowing	Significant shadowing	Significant shadowing	Significant shadowing	Minimal shadowing	Minimal shadowing	Minimal shadowing under silicone tip, significant under shaft	Significant shadowing	Significant shadowing under shaft, minimal under tip

Supplementary movie captions:

Supplementary Movie S1. 4D MIOCT of human retinal brushing with a membrane scraper. 4D MIOCT was performed during retinal brushing to initiate peeling of a pathologic membrane. Volumetric retinal deformation during the brushing maneuver and residual retinal deformation after the maneuver was only visible in the 4D MIOCT data.

Supplementary Movie S2. 4D MIOCT of human retinal brushing with a microsurgical flex loop. 4D MIOCT illustrating retinal brushing with a microsurgical flex loop and elevated blood vessels was performed during human eye surgery. 4D MIOCT revealed focal retinal depression during instrument contact.

Supplementary Movie S3. 4D MIOCT of enhanced visualization during removal of human pathologic translucent membranes. 4D MIOCT was performed during peeling of epiretinal membranes during human retinal surgery. The 4D MIOCT recording illustrates grasping and peeling of an ERM with surgical intraocular forceps and allowed detailed visualization of the membrane flap and peel angle.

Supplementary Movie S4. 4D MIOCT-guided identification and resolution of abnormal adhesion of iris to the corneal/graft interface. 4D MIOCT imaging was performed during a human full corneal transplant, and was necessary to identify and treat abnormal iris adhesion to the cornea/graft interface Using 4D MIOCT for localization guidance, the surgeon was able to direct a cannula and inject viscoelastic between the iris and corneal graft to release the adhesion. Further evaluation using MIOCT revealed resolution of the adhered iris with clear intervening space between iris and cornea.

Supplementary Movie S5. 4D MIOCT-guided unfolding of graft below native human cornea. 4D MIOCT imaging was performed during a human partial corneal transplant to visualize the axial distance between the graft and native cornea. As was corneal graft was unfolded under native cornea, the surgeon used 4D MIOCT to monitor the cornea/graft interface and confirm apposition. An artifact due to specular reflection from the corneal apex is present as a column in the middle of the volumes.
